# TangShenWeiNing formula alleviates diabetic kidney disease by inhibiting ferroptosis via AMPK pathway in renal tubular epithelial cells

**DOI:** 10.3389/fendo.2026.1749994

**Published:** 2026-02-12

**Authors:** Ye Jiang, Jing Chang, You Guo, Zhe Liu, Xiaomeng Feng

**Affiliations:** 1 Department of Endocrinology, Beijing Chao-Yang Hospital, Capital Medical University, Beijing, China; 2 Department of Internal Medicine, Beijing Chao-Yang Hospital, Capital Medical University, Beijing, China

**Keywords:** AMPK, diabetic kidney disease, ferroptosis, glutathione peroxidase 4, renal tubular epithelial cells, TangShenWeiNing formula

## Abstract

**Background:**

Diabetic kidney disease (DKD) is a common microvascular complication of diabetes mellitus. Ferroptosis in renal tubular epithelial cells is a new hotspot in elucidating underlying molecular mechanism and treatment of DKD. Studies have shown that tubular lesions occur early in DKD, and high glucose induces cellular oxidative stress and promotes ferroptosis in tubular epithelial cells. TangShenWeiNing (TSWN) formula shows a beneficial therapeutic effect for alleviating DKD, but its underlying molecular mechanism remains enigmatic. Previous studies have suggested that TSWN formula alleviates DKD by regulating adenylate-activated protein kinase (AMPK) pathway.

**Methods:**

The 8-week-old db/m and db/db mice were given low, medium, and high doses of TSWN as well as valsartan by gavage for 12 weeks, respectively.

**Results:**

Compared with non-diabetic mice, diabetic mice showed elevated urinary albumin, which was reduced by treatment with TSWN or valsartan. Intrarenal fibrosis and type I collagen expression were induced in diabetic mice compared to nondiabetic mice. TSWN or valsartan treatment attenuated these effects. Renal tubular injury was increased in diabetic mice, but TSWN or valsartan treatment ameliorated this damage. Diabetes led to elevated iron levels in the renal tubules of mice, which were attenuated after treatment with TSWN or valsartan. The levels of intrarenal p-AMPK/t-AMPK were reduced in db/db mice compared with db/m mice, and were increased by treatment with TSWN or valsartan in db/db mice. Diabetes aggravated reactive oxygen species (ROS) formation in the kidneys of db/db mice, and this increase was inhibited by TSWN or valsartan treatment. Renal gp47^phox^ was markedly upregulated in diabetic mice, whereas TSWN or valsartan administration significantly attenuated this elevation. Compared with db/m mice, db/db mice showed elevated renal malondialdehyde (MDA) and reduced expression of superoxide dismutase (SOD), catalase (CAT) and glutathione peroxidases (GSH-Px), and treatment with TSWN or valsartan lowered MDA while simultaneously restoring SOD, CAT and GSH-Px levels. Diabetic mouse kidneys exhibited marked downregulation of glutathione peroxidase 4, however, this reduction was reversed by TSWN formula or valsartan treatment in db/db mice.

**Conclusions:**

TSWN formula alleviated DKD by inhibiting ferroptosis via AMPK pathway in renal tubular epithelial cells.

## 1 Introduction

Diabetic kidney disease (DKD) is a common microvascular complication of diabetes mellitus, and is one of the major causes of end-stage renal disease (1, 2). Therefore, it is of great clinical and social significance to find effective measures to prevent DKD (3, 4). Recent increasing evidence has revealed that renal tubular injury occurs even in the early stages in DKD (5–8). Renal tubular injury, driven by oxidative stress, inflammation, hypoxia and fibrosis, promotes chronic kidney disease (CKD) progression, including DKD. (9–12). These findings indicate that renal tubular epithelial cells represent a significant therapeutic target in DKD.

Ferroptosis is a regulated form of cell death characterized by lipid peroxidation and plasma membrane damage (13). Renal biopsy studies reveal that patients with DKD exhibit elevated content of Fe²^+^ and down-regulation of the lipid-repair enzyme glutathione peroxidase 4. All of which correlate positively with proteinuria and inversely with estimated glomerular filtration rate (14). Besides, ample evidence has shown that ferroptosis drives rising urinary albumin levels, renal tubular damage and exacerbating renal fibrosis, whereas its inhibition alleviates diabetic kidney disease. (15, 16).

Adenylate-activated protein kinase (AMPK) plays a key role in regulating energy homeostasis in eukaryotes (17, 18). AMPK is activated when energy levels decrease. This activates pathways that produce adenosine triphosphate (ATP) and promote glucose uptake, while inhibiting ATP-depleting pathways associated with glucose synthesis. AMPK is turned on when its alpha subunit is phosphorylated at Thr172. AMPK activation widely affects glucose molecules and lipid metabolism by phosphorylated AMPK (pAMPK)-mediated phosphorylation of downstream target proteins (19). Ferroptosis is a form of regulated cell death directly executed by the lethal accumulation of lipid peroxides (20). The AMPK, a central regulator of cellular energy and metabolism, functions as an upstream checkpoint governing both lipid metabolism and the cellular antioxidant defense system (21–23). Consequently, AMPK activation exerts dual protective effects against ferroptosis. First, it modulates lipid metabolism by inhibiting fatty acid synthesis and promoting fatty acid β-oxidation, thereby reducing the pool of lipid substrates susceptible to peroxidation (24, 25). Second, it enhances the cellular capacity to eliminate lipid peroxides by upregulating key antioxidant proteins, such as glutathione peroxidase 4 (26, 27). Thus AMPK activation effectively prevents ferroptosis at its root. Substantial research progression indicates that AMPK exerts its inhibitory effect in the regulation of ferroptosis (26.

The treatment of DKD emphasizes comprehensive management, including simultaneous control of blood glucose, lipids and blood pressure, and the use of angiotensin-converting enzyme inhibitors/angiotensin receptor blockers (ACEI/ARB) in conjunction with chemical medications such as sodium glucose cotransporter 2 inhibitors (SGLT2i), glucagon-like peptide-1 receptor agonists (GLP1-RA), and nonsteroidal mineralocorticoid receptor antagonists (NS-MRA) to slow deterioration of renal function (28–32). However, satisfactory therapies may not be achieved using these conventional strategies of treating DKD.

Traditional Chinese medicine (TCM) has been extensively used in clinical and healthcare applications all over the world. Increasing evidence has revealed that TCM displays a beneficial effects for the treatment of DKD (33, 34). TangShenWeiNing (TSWN) has been clinically used to treat DKD for a long time, with a beneficial effect. Network pharmacology evidence suggests that TSWN formula has therapeutic effects on DKD, and its mechanism of underlying molecular mechanisms may be related to regulating AMPK signaling pathway (35), but the mechanism has been less explored. Previous studies have indicated that ferroptosis plays an important role in the development of DKD (36, 37). In this study, we aimed to investigate whether TSWN formula can alleviate DKD by regulating the AMPK pathway to inhibit ferroptosis in renal tubular epithelial cells of diabetic mouse models. This will provide new evidence to elucidate the renal protective mechanism of this medication and offer a potential therapeutic target for DKD.

## 2 Materials and methods

Experiments involving animals were approved by the Animal Ethics Committee of Beijing Chao-Yang Hospital, Capital Medical University, and conducted in accordance with the animal care guidelines of Beijing Chao-Yang Hospital, Capital Medical University.

### 2.1 Preparation of TSWN formula

TSWN (Patent No. 202111331292.3) was composed of 20g Huangqi (ASTRAGALI RADIX), 15g Taizishen (PSEUDOSTELLARIAE RADIX), 10g Danggui (ANGELICAE SINENSIS RADIX), 20g Dihuang (REHMANNIAE RADIX), 10g Shanzhuyu (CORNI FRUCTUS), 15g Shanyao (DIOSCOREAE RHIZOMA), 15g Tianhuafen (TRICHOSANTHIS RADIX), 15g Gouqizi (LYCII FRUCTUS), 15g Danshen (SALVIAE MILTIORRHIZAE RADIX ET RHIZOMA), 15g Fuling (PORIA), 10g Zexie (ALISMATIS RHIZOMA), 15g Taoren (PERSICAESE MEN), and 10g Gancao (GLYCYRRHIZAE RADIX ET RHIZOMA). TSWN was prepared and quality-controlled by Pharmacy of Chao-Yang Hospital, Capital Medical University. Previously, its chemical profile has been characterized by ultra-high performance liquid chromatography-tandem mass spectrometry (UHPLC-MS) analysis (35).

### 2.2 Intervention

Male C57BLKS/J db/m and db/db mice (t002407) (7 weeks old) were supplied by Nanjing Biomedical Research Institute of Nanjing University, Nanjing, China. They were rotationally assigned into db/m, db/db, db/db+V, db/db+TSWN-L, db/db+TSWN-M and db/db+TSWN-H groups, with 6 mice in each group. 185 g of TSWN were decocted by immersion in 400 ml of pure water at a concentration of 2 g/ml. db/m and db/db groups were given purified water twice daily. Mice in the db/db+V group received oral administration of valsartan (10.29mg/kg; Novartis Pharmaceuticals, Beijing, China), dissolved in purified water once daily, with the dosage based on the commonly administered dose for adults and purified water once daily. In this study, low, medium and high doses of 6.01, 12.02 and 24.05 g/kg were used in the db/db+TSWN-L, db/db+TSWN-M and db/db+TSWN-H groups respectively, twice a day (the medium dose was based on the commonly administered dose in adults). By supplementing pure water, the amount of gavage in each group was consistent with that of the db/db+TSWN-H group. All the above gavage treatments were carried out for 12 weeks from 8 weeks of age. The experimental conditions were: n = 6/cage; light cycle, 12h light/dark cycle (lights on 08:00 - 20:00 h); temperature: 22 ± 1°C; humidity, 40%; free water and food; and litter change, once a day. After 12 weeks of administration, animals were kept in separate metabolic cages to collect urine samples. At week 20, cardiac blood was collected via left-ventricular puncture and plasma was stored at −80°C. The mice were immediately euthanized, and tissue samples were harvested.

### 2.3 Urinary and blood parameters

Blood was collected following an overnight fast for 8h. Fasting blood glucose levels were measured using a HemoCue B-Glucose kit (HemoCue AB, Angelholm, Sweden). Creatinine in serum and urine levels were quantified by high performance liquid chromatography (Beckman Instruments, Fullerton, CA, USA). Urinary albumin levels were measured with immunoassay (Bayer, Elkhart, IN, USA). The urinary albumin to creatinine ratio (UACR) was expressed as urinary albumin/urine creatinine (mg/mg). Concentrations of kidney injury molecule-1 (KIM-1) and neutrophil gelatinase-associated lipocalcin (NGAL) in urine were measured by commercially available ELISA kits (R&D Systems, MN, USA). All procedures were conducted in strict accordance with the manufacturers’ instructions.

### 2.4 Light microscopy

Renal tissues were fixed in 10% formalin solution (SF93-20; Fisher Scientific, Pittsburgh, PA, USA). Paraffin sections of 8 µm thickness were prepared. Renal tubular injury was assessed using hexamine silver staining. After staining kidney tissue with hexamine silver, renal tubular injury was scored in six grades according to brush-border loss, tubular dilation, cast formation, necrosis, and neutrophil infiltration. Ten high-power fields were randomly selected-five in the cortex and five at the cortico-medullary junction each assigned 0–5 points: 0, normal; 1, mild (0-10%); 2, moderate (11-25%); 3, severe (26-49%); 4, highly severe (50-75%); 5, extensive (>75%). Two investigators blinded to group identity performed all evaluation (38). Masson staining was used to determine the degree of renal fibrosis. Iron content was determined using the Lillie staining method. Kidney specimens were embedded in optimal cutting temperature compound (4585; Fisher Health Care, Houston, TX, USA) and snap-frozen. Cryosections (8 µm) were cut and immediately incubated with dihydroethidium (DHE) to determine reactive oxygen species (ROS). Images were captured and quantified using ImageJ software (NIH, Bethesda, MD, USA). For light microscopy histochemistry, the sample size was n = 3 mice per group for Lillie staining, and n = 6 mice per group for both Masson’s trichrome and hexamine silver staining. From each animal’s renal cortex section, six random fields of view were selected under a 20× objective lens using a predefined grid pattern. The order of acquisition was determined by computer-generated random numbers, with observers unaware of the grouping to ensure unbiased sampling. Histopathological scoring (tubular injury score, fibrosis volume fraction, iron content fraction) was carried out by two independent investigators blinded to group allocation.

### 2.5 Western blot assay

Collect mouse kidney tissues and homogenize using a lysis buffer. The homogenates were subjected to centrifugation at 16,000×g for 15 min at 4°C. The bicinchoninic acid protein assay kit (Pierce Co., Rockford, IL, USA) was used to analyze protein concentrations. Equal amounts of each protein sample (20 µg) were resolved using 10% sodium dodecyl sulfate polyacrylamide gel electrophoresis (SDS-PAGE) and subsequently blotted onto a polyvinylidene difluoride (PVDF) membrane. After blocking in 5% non-fat milk in tris buffered saline, the blots were probed overnight with the following primary antibodies: Collagen I (1:10 00; Abcam, Cambridge, MA, USA), ferritin heavy chain (1:1000; Abcam, Cambridge, MA, USA), t-AMPK(1:1000; Cell Signaling Technologies, Boston, MA, USA), p-AMPK(1:1000; Cell Signaling Technologies, Boston, MA, USA), gp47^phox^ (1:1000; BD transduction, San Jose, CA USA), glutathione peroxidase 4 (GPX4) (1:1000; Abcam, Cambridge, MA, USA) and β-actin (1:1000; Cell Signaling, Danforth, MA, USA). After washing, membranes were incubated with horseradish peroxidase-coupled secondary antibody (1:5000; Santa Cruz, CA, USA) for 2 hours. Density measurements were analyzed using image acquisition and analysis software (Bio-Rad).

### 2.6 Evaluation of oxidative stress indicators in mouse renal tissues

In accordance with the manufacturer’s operating procedures, commercial kits (Beyotime Institute of Biotechnology, Shanghai, China) were employed to determine malondialdehyde (MDA) in renal tissue homogenates using the thiobarbituric acid method, the superoxide dismutase (SOD) using the xanthine oxidase method, glutathione peroxidases (GSH-Px) using the nicotinamide adenine dinucleotide phosphate (NADPH) method, and catalase (CAT) using the coloration method.

### 2.7 Statistical analysis

Statistical analysis was performed using GraphPad Prism 9 (GraphPad Software, San Diego, CA, USA). Data were expressed as mean ± SD, and comparisons between multiple groups were analyzed by one-way ANOVA, with a P value of <0.05 considered significant.

## 3 Results

### 3.1 Physical, biochemical and renal functional parameters of mice

**Figure 1:**
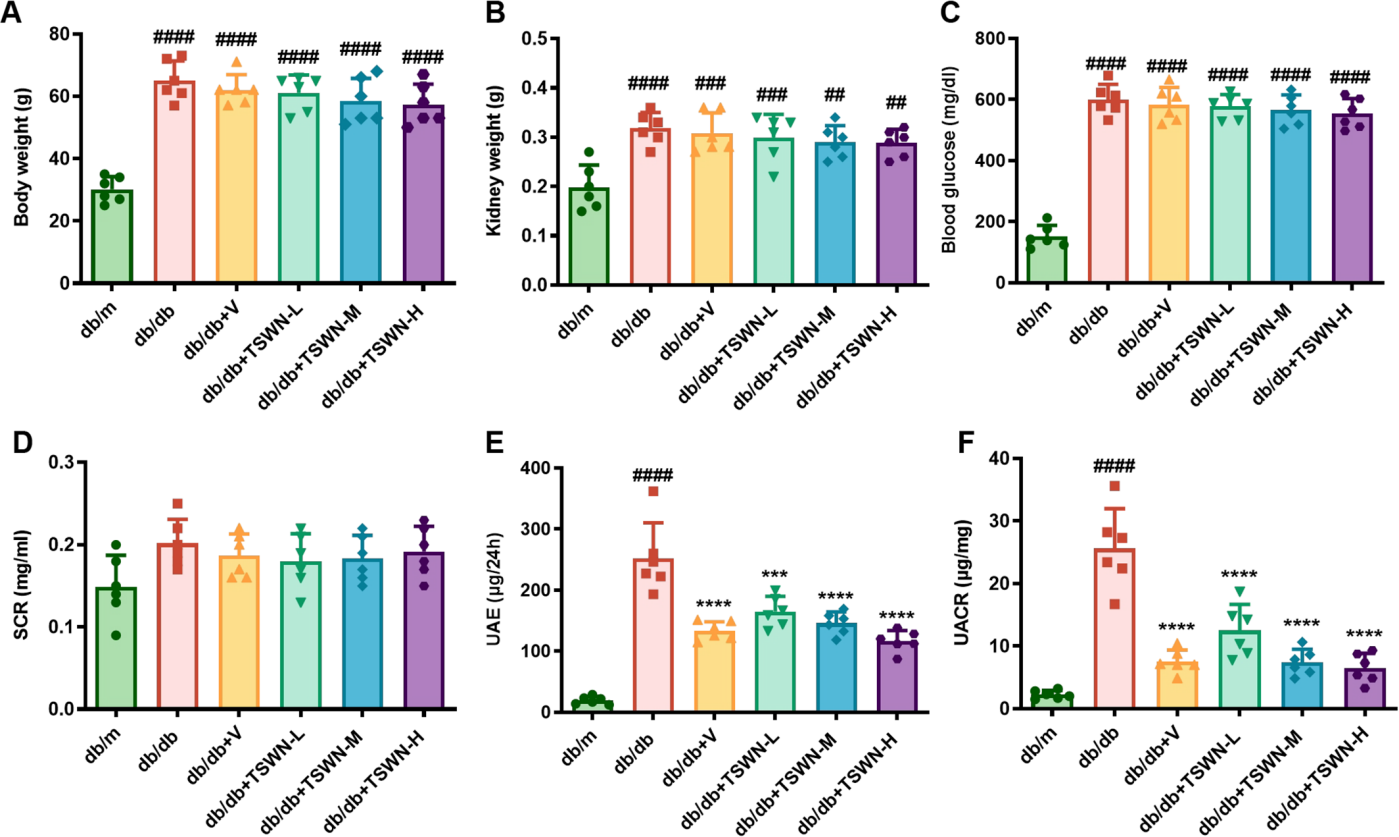
Physical, biochemical and renal functional parameters of mice. **(A–C)** Body weight, kidney weight, and blood glucose levels in db/m, db/db, and db/db mice treated with valsartan or different doses of TSWN. **(D–F)** SCr, UAE, and UACR in db/m, db/db, and db/db mice treated with valsartan or different doses of TSWN. n = 6 mice/group. ^##^P < 0.01, ^###^P < 0.001, ^####^P<0.0001 vs db/m group; ^***^P < 0.001, ^****^P < 0.0001 vs db/db group. Db/m, db/m mice; db/db, db/db mice; db/db+V, db/db mice with valsartan treatment; db/db+TSWN-L, db/db mice with low dose TSWN treatment; db/db+TSWN-M, db/db mice with middle dose TSWN treatment; db/db+TSWN-H, db/db mice with high dose TSWN treatment.

As shown in **Figures 1A–C**, compared with the db/m group, body weight, kidney weight and blood glucose levels were remarkably elevated in the db/db, db/db+V, db/db+TSWN-L, db/db+TSWN-M, and db/db+TSWN-H groups. However, there were no significant differences in body weight, kidney weight, and blood glucose levels between db/db mice treated with or without TSWN formula or valsartan. As demonstrated in **Figure 1D**, serum creatinine (SCR) levels were similar across all six groups of mice. As shown in **Figures 1E, F**, compared to the db/m group, the db/db group exhibited elevated urinary albumin excretion (UAE) and UACR. Additionally, compared with the db/db group, the db/db+V group, db/db+TSWN-L group, db/db+TSWN-M group, and db/db+TSWN-H group all exhibited significantly reduced levels of UAE and UACR. This suggests that TSWN or valsartan treatment reduced urinary albumin in diabetic mice.

### 3.2 Determination of renal fibrosis

**Figure 2:**
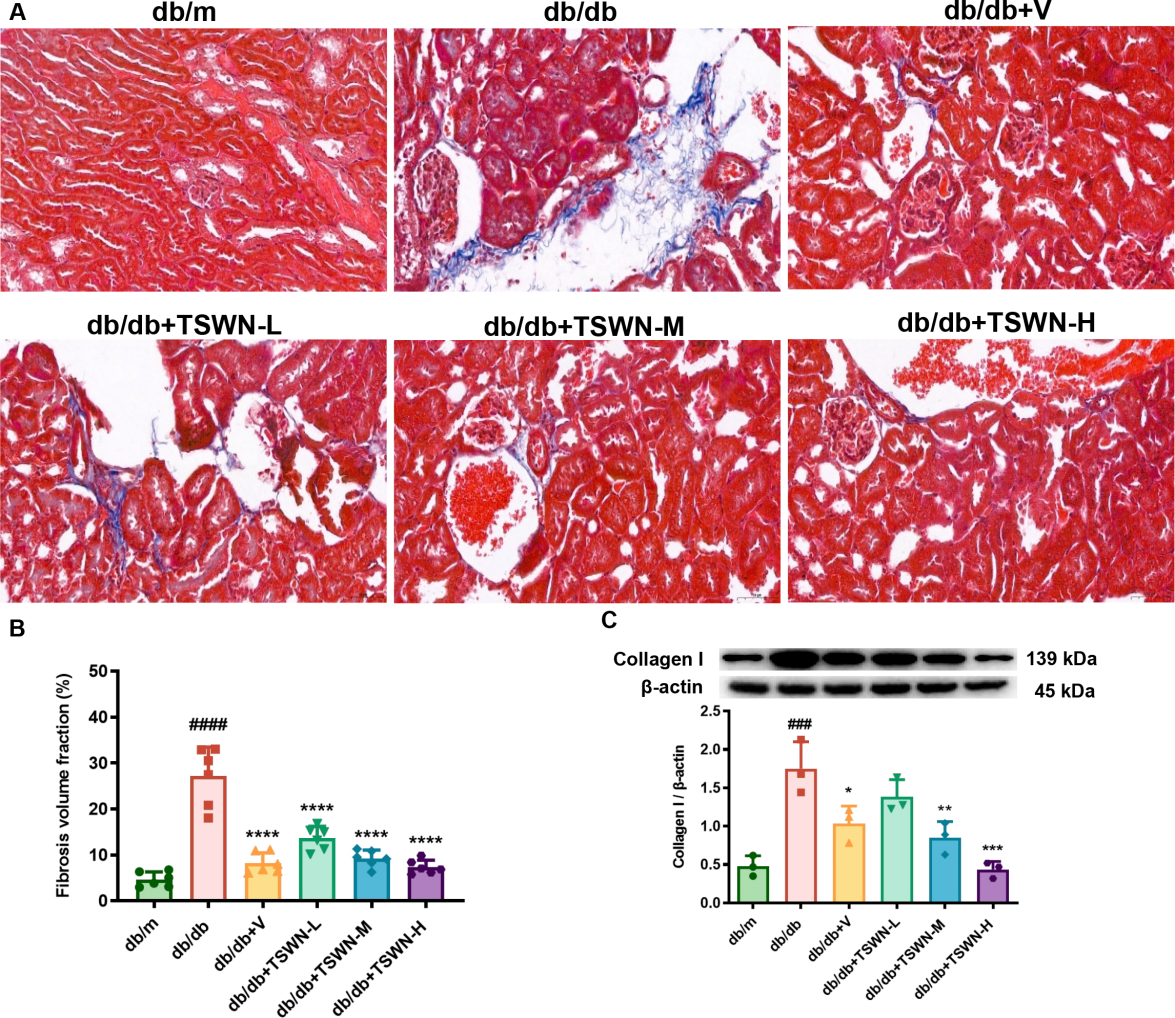
TSWN formula or valsartan treatment inhibited renal fibrosis in diabetic mice. **(A)** Images of Masson’s staining in the kidney tissues of db/m, db/db, and db/db mice treated with valsartan or different doses of TSWN. **(B)** Quantification of renal fibrosis in the kidney tissues of db/m, db/db, and db/db mice treated with valsartan or different doses of TSWN. **(C)** Protein expression and quantitative analyses of collagen I in the kidney tissues of db/m, db/db, and db/db mice treated with valsartan or different doses of TSWN. n =3–6 mice/group. ^###^P < 0.001, ^####^P<0.0001 vs db/m group; ^*^P < 0.05, ^**^P < 0.01, ^***^P < 0.001, ^****^P < 0.0001 vs db/db group. Db/m, db/m mice; db/db, db/db mice; db/db+V, db/db mice with valsartan treatment; db/db+TSWN-L, db/db mice with low dose TSWN treatment; db/db +TSWN-M, db/db mice with middle dose TSWN treatment; db/db+TSWN-H, db/db mice with high dose TSWN treatment.

Masson’s staining was employed to evaluate the extent of renal fibrosis (**Figures 2A, B**). The results revealed that diabetes markedly exacerbated renal fibrosis in mice, while TSWN or valsartan inhibited renal fibrosis in diabetic mice. Western blot analysis further demonstrated that diabetes increased the expression of collagen I, a protein associated with renal fibrosis, while treatment with TSWN or valsartan inhibited its expression in diabetic mice (**Figure 2C**).

### 3.3 Determination of renal tubular injury

**Figure 3:**
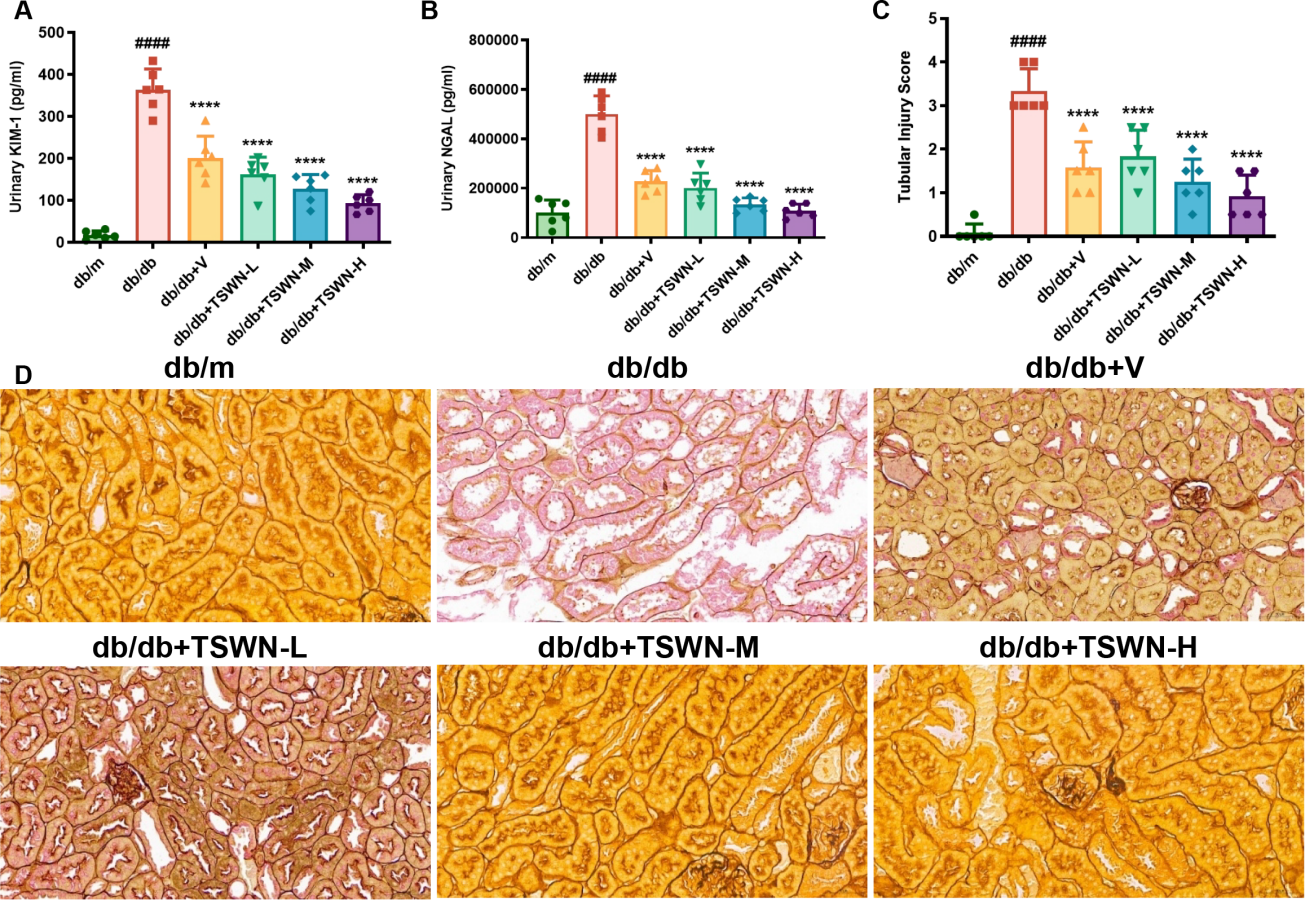
TSWN formula or valsartan treatment alleviated renal tubular injury of db/db mice. **(A, B)** Quantification of urinary KIM-1 and NGAL in the kidney tissues of db/m, db/db, and db/db mice treated with valsartan or different doses of TSWN. by ELISA. **(C)** Quantification of tubular injury score in the kidney tissues of db/m, db/db, and db/db mice treated with valsartan or different doses of TSWN. **(D)** Images of hexamine silver staining of tubular injury in the kidney tissues of db/m, db/db, and db/db mice treated with valsartan or different doses of TSWN. n = 6 mice/group. ^####^P < 0.0001 vs db/m group; ^****^P < 0.0001 vs db/db group. Db/m, db/m mice; db/db, db/db mice; db/db+V, db/db mice with valsartan treatment; db/db+TSWN-L, db/db mice with low dose TSWN treatment; db/db +TSWN-M, db/db mice with middle dose TSWN treatment; db/db+TSWN-H, db/db mice with high dose TSWN treatment.

Hyperglycemia is a major metabolic stressor in diabetes mellitus and is considered as an important causative factor in the development of DKD (39, 40). Hyperglycemia promotes oxidative stress and fibrosis, and the severity of renal tubular injury correlates with the amount of proteinuria in patients with DKD (41, 42). The KIM-1 and NGAL are important biomarkers of renal tubular injury (43). To evaluate renal tubular injury in diabetic mice, the levels of KIM-1 and NGAL were measured as shown in **Figures 3A, B**, compared with db/m controls, db/db mice exhibited significantly elevated levels of KIM-1 and NGAL. These increases were substantially reduced by treatment with TSWN or valsartan. Moreover, hexamine silver staining revealed that diabetes caused folding and layering of the tubular basement membrane, whereas treatment with TSWN formula or valsartan alleviated the damage to the tubular basement membrane in db/db mice (**Figure 3D**). The renal tubular injury score increased in db/db mice, and treatment with TSWN formula or valsartan alleviated renal tubular injury (**Figures 3C, D**).

### 3.4 Iron accumulation in the kidney of mice

**Figure 4:**
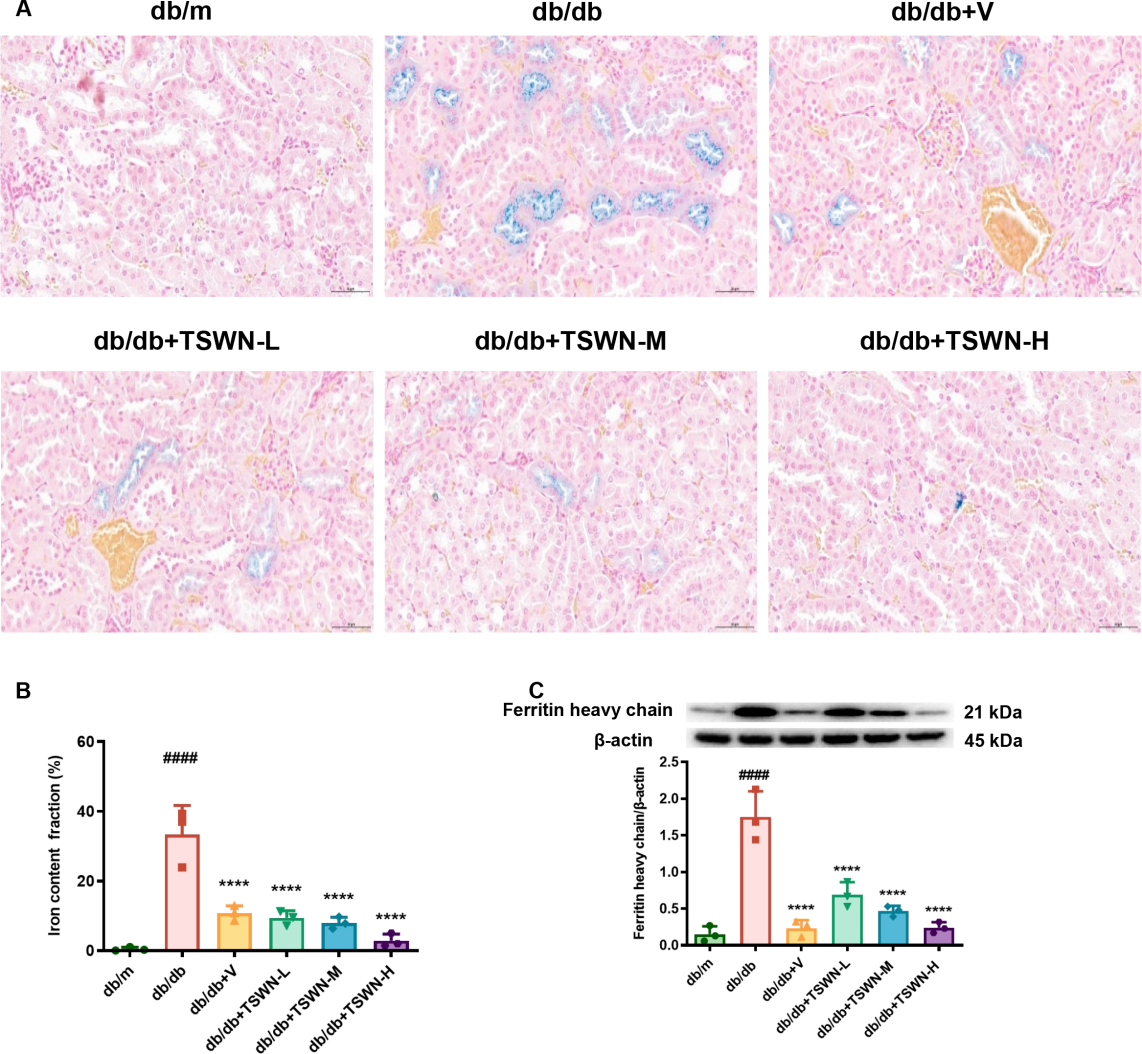
TSWN formula or valsartan treatment decreased iron levels in the kidneys of db/db mice. **(A)** Images of Lillie staining of iron content (blue) in the kidney tissues of db/m, db/db, and db/db mice treated with valsartan or different doses of TSWN. **(B)** Quantification of iron content in the kidney tissues of db/m, db/db, and db/db mice treated with valsartan or different doses of TSWN. **(C)** Protein expression and quantitative analyses of ferritin heavy chain in the kidney tissues of db/m, db/db, and db/db mice treated with valsartan or different doses of TSWN. n = 3 mice/group. ^####^P < 0.0001 vs db/m group; ^****^P < 0.0001 vs db/db group. Db/m, db/m mice; db/db, db/db mice; db/db+V, db/db mice with valsartan treatment; db/db+TSWN-L, db/db mice with low dose TSWN treatment; db/db +TSWN-M, db/db mice with middle dose TSWN treatment; db/db+TSWN-H, db/db mice with high dose TSWN treatment.

Iron is required for a large number of metabolic enzymes in the generation of cellular ROS. However, iron overload can lead to the accumulation of free iron, exacerbating ROS production and triggering oxidative stress. Ferroptosis is triggered when oxidative stress exceeds the cell’s repair capacity, along with glutathione (GSH) depletion, inhibition of GPX4 activity, and accumulation of lipid peroxidation. Ferroptosis is an iron-dependent form of cell death (44). Consequently, renal iron deposition in diabetic mice was evaluated. As shown in **Figures 4A, B**, Lillie staining revealed markedly elevated tubular iron levels in diabetic mice, an effect that was substantially reversed by either TSWN or valsartan treatment. The results of the Western blot analysis demonstrated an increased expression of ferritin heavy chain in the kidneys of db/db mice in comparison with db/m mice. Conversely, TSWN or valsartan treatment resulted in a decreased expression of ferritin heavy chain (**Figure 4C**). This suggests that diabetes increases iron levels in the renal tubules and promotes ferroptosis in renal tubular epithelial cells, while TSWN or valsartan treatment attenuates this effect.

### 3.5 Renal AMPK activation, oxidative stress, lipid peroxidation and GPX4 levels

**Figure 5:**
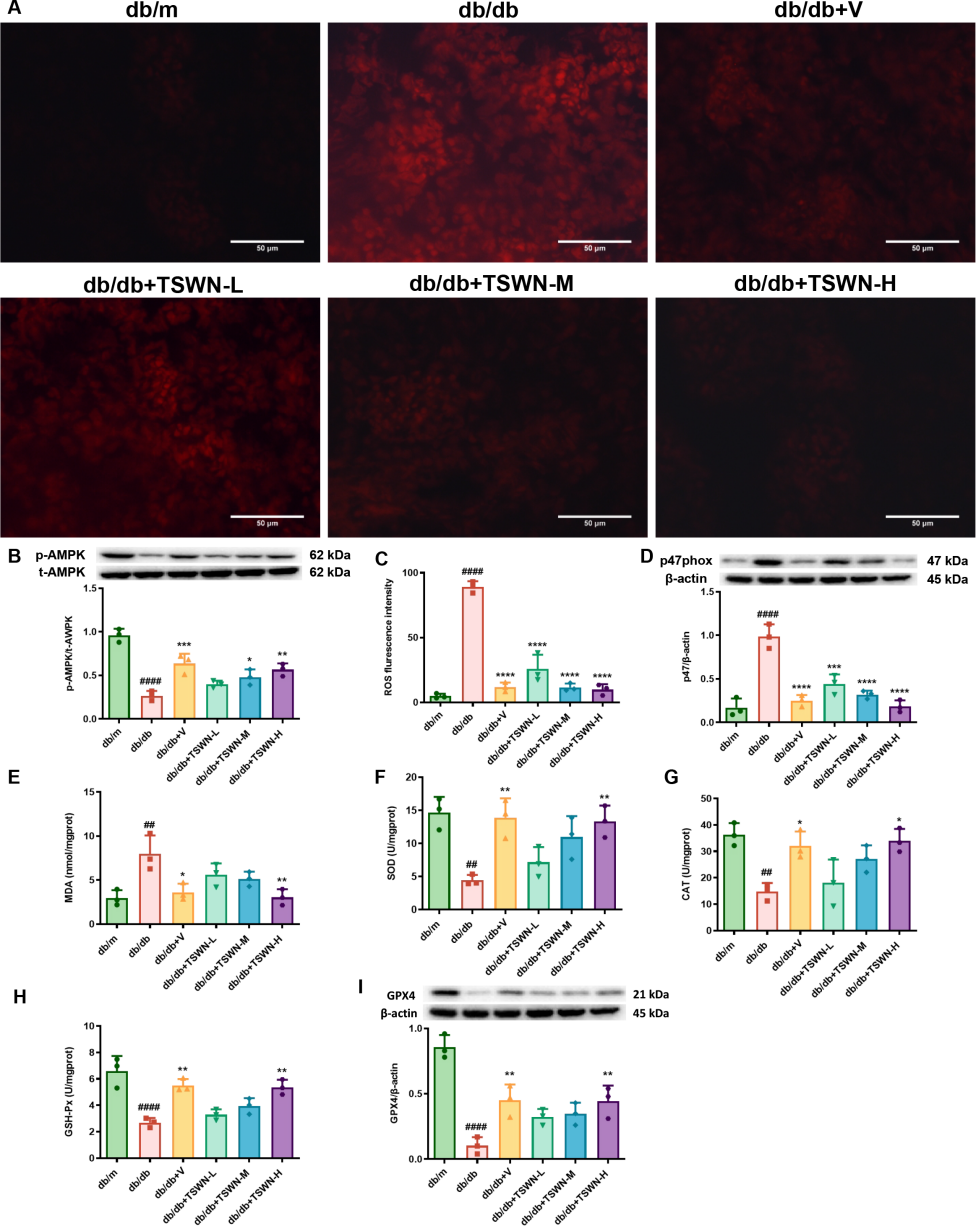
TSWN formula or valsartan treatment elevated the p-AMPK/t-AMPK ratio, ameliorated oxidative stress and lipid peroxidation and raised GPX4 levels in the kidneys of db/db mice. **(A)** Images of dihydroethidium staining of ROS formation (red) in the kidney tissues of db/m, db/db, and db/db mice treated with valsartan or different doses of TSWN. **(B)** Protein expression and quantitative analyses of p-AMPK/t-AMPK in the kidney tissues of db/m, db/db, and db/db mice treated with valsartan or different doses of TSWN. **(C)** Quantification of ROS formation in the kidney tissues of db/m, db/db, and db/db mice treated with valsartan or different doses of TSWN. **(D)** Protein expression and quantitative analyses of gp47^phox^ in the kidney tissues of db/m, db/db, and db/db mice treated with valsartan or different doses of TSWN. **(E–H)** Quantification of MDA, SOD, CAT and GSH-Px in the kidney tissues of db/m, db/db, and db/db mice treated with valsartan or different doses of TSWN. **(I)** Protein expression and quantitative analyses of GPX4 in the kidney tissues of db/m, db/db, and db/db mice treated with valsartan or different doses of TSWN. n = 3 mice/group. ^##^P < 0.01, ^####^P < 0.0001 vs db/m group; ^*^P < 0.05, ^**^P < 0.01, ^***^P < 0.001, ^****^P < 0.0001 vs db/db group. Db/m, db/m mice; db/db, db/db mice; db/db+V, db/db mice with valsartan treatment; db/db+TSWN-L, db/db mice with low dose TSWN treatment; db/db+TSWN-M, db/db mice with middle dose TSWN treatment; db/db+TSWN-H, db/db mice with high dose TSWN treatment.

When glucose is absent, AMPK is activated to initiate an energy stress protection program that counteracts ferroptosis (44). As shown in **Figure 5B**, western blot revealed that the p-AMPK/t-AMPK ratio was reduced in the kidneys of db/db mice compared to db/m mice, while treatment with TSWN formula or valsartan elevated the p-AMPK/t-AMPK ratio in the kidneys of db/db mice. As demonstrated in **Figures 5A–D**, dihydroethidium staining revealed that diabetes exacerbated the formation of ROS in mouse kidneys, while TSWN or valsartan treatment inhibited ROS formation in db/db mouse kidneys. Diabetes caused an increase in the expression of NADPH oxidase subunit-gp47^phox^ in mouse kidneys, while TSWN or valsartan treatment reduced gp47^phox^ in db/db mouse kidneys. The findings indicated that diabetes facilitated the generation of ROS derived from NADPH oxidase in mouse kidneys, whereas treatment with TSWN or valsartan suppressed the ROS generation in diabetic mouse kidneys. As shown in **Figures 5E–H**, compared with the db/m group, MDA expression increased in the db/db group, while SOD, CAT, and GSH-PX expression decreased. Treatment with TSWN or valsartan led to a decrease in MDA expression and an increase in SOD, CAD, and GSH-PX expression. As shown in **Figure 5I**, GPX4 is a pivotal regulator of ferroptosis, converting lipid hydroperoxides into lipid alcohols and thereby blocking Fe²^+^-dependent lipid ROS accumulation. Loss of GPX4 activity has been demonstrated to induce lipid peroxidation and can trigger ferroptosis (45). We measured the expression of GPX4 in mouse kidneys. Western blotting revealed that GPX4 levels were markedly diminished in kidneys of diabetic mice, whereas either TSWN or valsartan treatment raised GPX4 levels. These results further suggested that ferroptosis driven by lipid peroxidation contributed to DKD, and that TSWN formula or valsartan can reduce lipid peroxidation, thereby improving DKD.

## 4 Discussion

This study showed that elevated levels of UAE and UACR were present in diabetic mice. Diabetes mellitus accelerated renal pathological changes in these mice, promoted renal fibrosis, and induced renal tubular injury. In addition, compared with non-diabetic mouse kidneys, diabetic mouse kidneys showed increased iron content, decreased p-AMPK/t-AMPK, increased ROS formation, increased expression of NADPH oxidase subunit-gp47^phox^, increased MDA expression, and decreased expression of SOD, CAT, and GSH-PX, as well as decreased expression of GPX4. However, TSWN formula can reduce UAE and UACR in diabetic mice, improve renal pathological changes, alleviate renal fibrosis, and improve renal tubular injury. Based on this, TSWN formula regulated the AMPK pathway in the renal tubules of diabetic mice, inhibited ferroptosis, and alleviated DKD.

DKD is a common microvascular complication of diabetes that can lead to renal failure in diabetic mellitus patients (46–48). Currently, treatment methods for DKD have not achieved satisfactory results (49). TCM has advantages such as multiple pathways and multiple targets (50). As an important therapy for CKD, TCM has been long practiced and demonstrated to be good clinical efficacy that significantly improved CKD patients and enhance their life quality (51–54). Recent studies have focused on identifying active components in TCM that protected against kidney injury and their underlying molecular mechanisms (55–57). Among these, the regulation of glycolipid metabolism, antioxidant, anti-inflammatory, anti-fibrotic, and podocyte protective effects have been identified as key pathways (58–61). Recent research into the pathogenesis of DKD has moved beyond traditional theories of hyperglycemia and hemodynamics, with increasing focus on the interplay between iron homeostasis imbalance and regulated cell death. A recent review systematically synthesized three lines of evidence supporting the critical role of ferroptosis in DKD (62). Firstly, significant abnormal deposition of ferrous iron (Fe²^+^) has been observed in renal biopsy samples from early-stage DKD patients and in animal models, with its level showing a significant positive correlation with the severity of clinical proteinuria, suggesting iron overload is an early pathological feature of DKD. Secondly, in renal tubular epithelial cells, the downregulation of the core antioxidant enzyme GPX4 directly leads to the collapse of the repair capacity for membrane phospholipid peroxidation, triggering a lethal burst of lipid ROS that drives ferroptosis. Finally, interventional studies confirm that the use of iron chelators or GPX4 activators can effectively attenuate renal fibrosis and abnormal deposition of extracellular matrix in DKD models (38, 62). This evidence collectively indicates a synergistic effect between ferroptosis and impaired glucose metabolism in DKD. The hyperglycemic environment sensitizes renal cells to ferroptosis through various pathways. Meanwhile, the intense oxidative stress generated by the ferroptosis process further damages neighboring cells and activates pro-fibrotic pathways, creating a vicious cycle that exacerbates renal function loss (63, 64).

TSWN formula is a kind of traditional Chinese herbal formulation comprising thirteen medicinal herbs. Research indicated that TSWN Formula played an important role in the treatment of DKD (10). Our current research indicated that UAE and UACR levels in diabetic mice were higher than those in control mice. However, treatment with the TSWN formula reduced UAE and UACR levels in diabetic mice, suggesting that the TSWN formula may improve DKD. Hyperglycemia is the primary metabolic stressor in diabetes and is considered as a significant pathogenic factor in the development and progression of DKD. Hyperglycemia promotes fibrosis in renal tubular epithelial cells (65). In this study, diabetic mice developed renal fibrosis with elevated renal collagen I content, whereas TSWN formula alleviated both changes, indicating its antifibrotic efficacy in DKD.

Renal tubular injury was evaluated in diabetic mice. Compared with non-diabetic controls, diabetic mice exhibited markedly elevated renal tubular injury biomarkers KIM-1 and NGAL in both renal tissue and urine, accompanied by pronounced folding and disruption of the tubular basement membrane (46). Nevertheless, the TSWN formula markedly alleviated these tubular lesions in diabetic mice, thereby indicating that it can effectively protect against tubular damage in DKD.

Iron is an essential element for the survival of cells (62). As a key cofactor for multiple redox enzymes, iron plays a dual role in cellular ROS metabolism (62). At physiological concentrations, iron ions participate in normal redox reactions. However, when free ferrous ions accumulate excessively within cells, large amounts of ROS are produced, disrupting the redox balance and inducing oxidative stress, thereby promoting ferroptosis, which has been implicated in the pathogenesis of numerous diseases (44, 66). Ferroptosis is closely related to renal ischemic injury (20). In the current study, Lillie staining revealed that diabetic mice exhibited augmented iron content in the renal tubules when compared with non-diabetic mice, but after treatment with TSWN formula, the iron content in the renal tubules of diabetic mice decreased. Western blot results showed that, compared with non-diabetic mice, diabetic mice had elevated ferritin heavy chains in their kidneys, while treatment with TSWN formula reduced ferritin heavy chains in the kidneys of diabetic mice. The above results indicated that diabetes caused iron overload in the renal tubules of mice. However, the administration of the TSWN formula resulted in a reduction of iron accumulation in the renal tubules of diabetic mice. Furthermore, the TSWN formula inhibited ferroptosis, thereby reducing renal tubular injury in diabetic mice.

On the basis of mass spectrometry analysis and network pharmacology research, TSWN formula may prevent DKD and decrease urinary albumin levels by AMPK pathway (35). Studies have shown that when glucose is excessive, AMPK activity is low and lipid peroxidation accumulates, thereby promoting ferroptosis (44). AMPK has an inhibitory effect in regulating ferroptosis, and activating AMPK may improve diseases or pathological conditions caused by ferroptosis (26). In this study, compared with non-diabetic mice, diabetic mice had lower p-AMPK/t-AMPK levels in kidneys. In contrast, treatment with TSWN formula increased p-AMPK/t-AMPK levels in kidneys of diabetic mice. This suggests that TSWN formula alleviates DKD through AMPK pathway. While our data show that TSWN increases AMPK phosphorylation, we acknowledge that these findings are correlative. Definitive proof that AMPK activation is necessary and sufficient for TSWN-mediated ferroptosis inhibition will require tubule-specific AMPK knockout or pharmacological blockade experiments, which are planned in our follow-up project. Beyond the ferroptosis pathway dissected here, AMPK is increasingly recognized as a multifunctional metabolic checkpoint that simultaneously governs autophagy, cellular senescence and systemic glucose-lipid homeostasis (67, 68). Whether TSWN engages these additional arms to reinforce renal protection remains unexplored.

Ferroptosis regulates cell death characterized by iron-dependent lipid peroxidation (69). ROS-induced lipid peroxidation plays a key role in cell death, such as ferroptosis (70, 71). Excessive ROS induces lipid peroxidation through multiple pathways, leading to cell death (72, 73). NADPH oxidase is one of the important enzyme systems for ROS generation in cells (74). The role of excessively elevated ROS, caused by hyperglycemia, in the development of DKD is well established (75). Recent findings revealed that diabetes markedly elevated ROS generation in mouse kidneys, while treatment with TSWN formula reduced ROS formation in diabetic mouse kidneys. Compared to non-diabetic mouse kidneys, the expression of the NADPH oxidase subunit gp47^phox^ was significantly increased in diabetic mouse kidneys, and treatment with the TSWN formula inhibited the expression of gp47^phox^ in diabetic mouse kidneys. These data implied that diabetes boosted NADPH-oxidase-dependent ROS production in the kidney, whereas the TSWN formula suppressed this ROS generation by blocking ferroptosis in diabetic mice.

MDA is one of the primary end products of lipid peroxidation, and is commonly used as an indicator of oxidative stress and cellular damage (76–78). Hyperglycemia promotes ROS production, leading to elevated MDA levels in kidney tissue (79). SOD, CAT, and GSH-Px constitute a cascade antioxidant defense system (80).GPX4 has been uniquely recognized for its role in blocking ferroptosis through suppressing phospholipid peroxidation (81, 82). In our research, DKD mice showed elevated MDA and reduced SOD, CAT and GSH-Px, indicating severe oxidative stress. TSWN reversed these changes and restored GPX4 levels, thus suppressing lipid peroxidation and ferroptosis in diabetic kidneys. The present study provides the proof-of-concept that the AMPK/ferroptosis axis is operative in early diabetic kidney disease and that the TSWN formula can modulate this pathway. Nevertheless, formal demonstration of causality awaits the following functional rescue experiments, including ferroptosis inhibitor (Fer-1) or inducer (Erastin) intervention to verify that TSWN protection is truly ferroptosis-dependent and *in vitro* AMPK knock-down or dominant-negative mutation to confirm that GPX4 up-regulation is AMPK-dependent. These investigations need to be pursued in a follow-up project with larger sample sizes and multi-center validation. In addition, SA-β-Gal staining was not performed in this study, so direct quantification of cellular senescence in renal tubules is lacking and future work will include this assay. Before any therapeutic recommendation can be made, the efficacy and safety of TSWN must be confirmed in randomized, controlled clinical trials. Although our work shows that TSWN can suppress ferroptosis in diabetic kidney disease and that this effect is associated with AMPK activation, the causal dependence remains to be established. The precise mechanisms by which the entire TSWN formula exert renal protection therefore warrant further investigation.

## 5 Conclusions

In conclusion, our research indicated that TSWN provides a candidate intervention strategy for diabetic kidney disease. It reduced urinary albumin in diabetic mice, improved renal tissue damage, alleviated renal fibrosis, reduced iron content in the renal tubules, increased p-AMPK/t-AMPK levels in the kidneys, inhibited the formation of ROS in the kidneys, reduced the expression of gp47^phox^ in the kidneys, leading to decreased MDA expression and increased SOD, CAT and GSH-PX expression, and increased the expression of GPX4 in the kidneys. TSWN formula inhibited ferroptosis in renal tubular epithelial cells through the AMPK pathway, thereby alleviating DKD.

## Data Availability

The raw data supporting the conclusions of this article will be made available by the authors, without undue reservation.
